# First person – Agamani Ghosal

**DOI:** 10.1242/bio.054163

**Published:** 2020-07-21

## Abstract

First Person is a series of interviews with the first authors of a selection of papers published in Biology Open, helping early-career researchers promote themselves alongside their papers. Agamani Ghosal is first author on ‘Communication between Cyclin dependent kinase Cdc2 and the Wis1-Spc1 MAPK pathway determines mitotic timing in *Schizosaccharomyces pombe*’, published in BiO. Agamani is a PhD student in the Cell Division lab of Dr Geetanjali Sundaram.


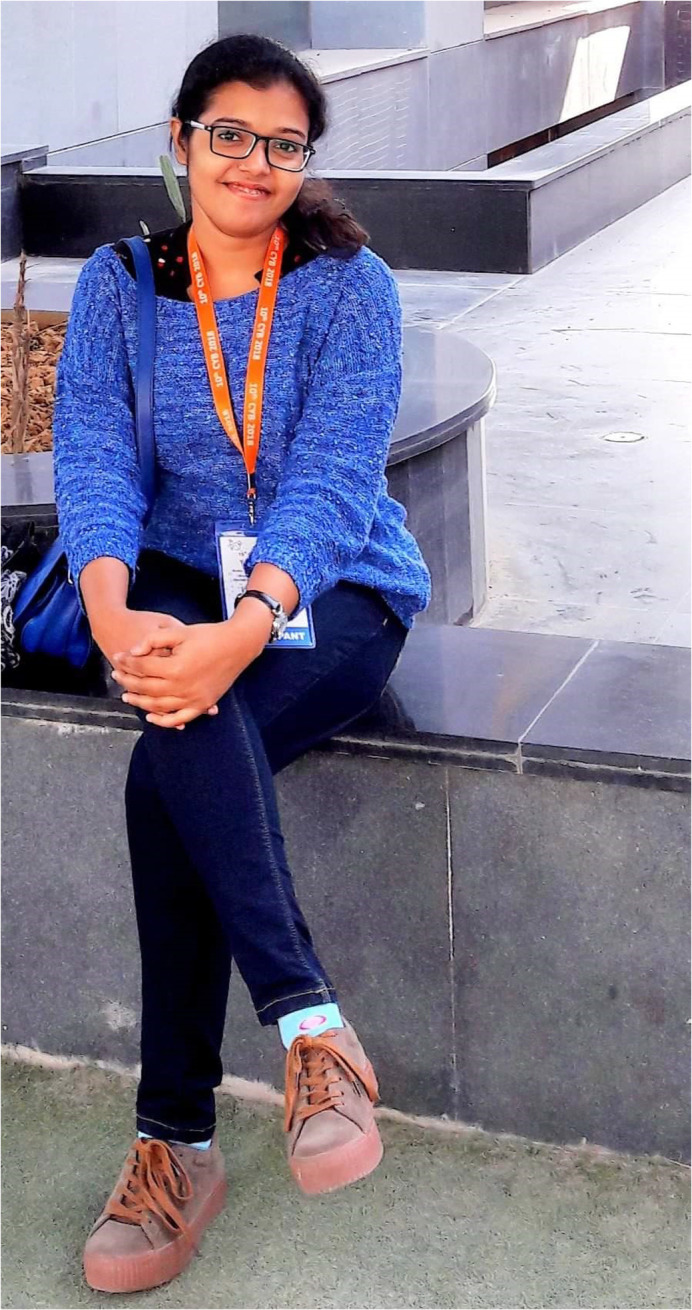


**Agamani Ghosal**

**What is your scientific background and the general focus of your lab?**

The long-held urge to pursue biology – after completing a degree in chemistry (honours) from Maulana Azad College, University of Calcutta – led me along the path to take up biochemistry for my masters from the University of Kalyani. My enthusiasm to pursue research has remained at the peak since then, as the senior scholars working in the department have been a true source of inspiration. I can't forget to mention Dr Swarup Roy, the senior scholar who guided me through my dissertation project in the Department of Biochemistry and Biophysics at the University of Kalyani and sort of implanted the seeds of a quest in me. It was destiny that gifted me the opportunity to follow my dreams by means of the DST-Inspire Fellowship and I landed up at the University of Calcutta, under the supervision of Dr Geetanjali Sundaram and joint supervision of Dr Paltu Kumar Dhal at Jadavpur University. The lab where I have already spent 5 years of my doctoral research mainly focuses on the regulation of cell division using fission yeast, *Schizosaccharomyces pombe*, as a model system. The two distinct protein targets we work with are Atf1 and Spc1 (I work with the latter) in order to delve in deeper into the regulatory roles they play in controlling mitosis.

**How would you explain the main findings of your paper to non-scientific family and friends?**

We work with a very interesting protein Spc1 (MAP kinase) whose human homolog, p38, is an implicated target in cancer therapy. The cell-division cycle is mainly controlled by the core regulator Cdc2 (cyclin-dependent kinase) in *S. pombe.* It is known that numerous adverse conditions, such as stress, lead to activation of the stress-activated MAPK Spc1, which in turn takes over the control of mitotic progression, thereby playing the role of a saviour. In other words, Spc1 emerges as the mighty hero of my story, as it is essentially required for cellular survival during stress. This is a long-established pathway, but whether changes in Cdc2 function can consequently impact on Spc1 activation remained unknown. We completely deshrouded this darkness when we found that an increase or decrease in Cdc2 function is simultaneously associated with an increase or decrease in the level of active Spc1. The fact that a decrease in Cdc2 activity is associated with decrease in Spc1 phosphorylation, both in the absence (being a reversible phenomenon) and presence of stimuli, suggests that a direct or indirect mode of communication between the two kinases exists. This mechanism was found to be dependent on Wis1 (the elderly right above Spc1 in the MAPK activation loop) as Cdc2 hyperactivation would not enhance Spc1 phosphorylation in absence of Wis1. Moreover, this feedback loop was also found to target another effector protein, Rad24 (a 14-3-3 homolog), which when upregulated tends to provide some protection to cells during Cdc2 hyperactivation. We also showed that replication stress can lead to increased Spc1 activity in a Wis1-dependent manner, and that inhibition of Spc1 can abrogate G1/S checkpoint activation.

“Spc1 emerges as the mighty hero of my story, as it is essentially required for cellular survival during stress.”

**What are the potential implications of these results for your field of research?**

We show for the first time a reciprocal influence of Cdc2 on phosphorylation and activation of Spc1, which can dictate mitotic timing even in absence of stress stimuli. Our findings identify Cdc2 hyperactivation and nucleotide depletion as novel triggers for Spc1 (MAPK) activation, thereby adding to the importance of Spc1 during stress response. Also, Rad24 levels were found to be in direct correlation with Spc1 activity, clearly claiming its place as a major effector involved in the communication between Spc1 and Cdc2. These very new findings add a novel perspective to our understanding of mitotic regulation by MAPKs and opens up prospects for research on CDK-driven regulation of MAPKs.

**What has surprised you the most while conducting your research?**

Earlier we had reported (Paul et al., 2018) that Spc1 overexpression or increased Spc1 activity due to oxidative stress can lead to increased *rad24^+^* expression in Δ*wee1* cells (with a hyperactive Cdc2) and not in wild-type *S. pombe* cells. The first striking observation we made while looking for the differences in the levels of Spc1 activity between the two was that phosphorylated Spc1 or active Spc1 was already higher, even in unstressed Δ*wee1* cells. This contributed to forming the initial base of our story. Our hints regarding the possibility of an existing connection (direct or indirect) between Cdc2 and Spc1 was quite a primitive one. This became foolproof when we found that reducing Cdc2 activity can in turn decrease Spc1 phosphorylation and that it was a completely reversible process. Moreover, the fact that complete Cdc2 inactivation at a non-permissive temperature fails to activate Spc1 even after oxidative stress was the most remarkable discovery we made while conducting this study.

**What, in your opinion, are some of the greatest achievements in your field and how has this influenced your research?**

An extensive survey will already give immense literary insight into the regulation of mitotic timing in fission yeast. Spc1 has been shown to be activated during stress and promote cellular survival through the adaptive stress response. Available reports suggest that low to moderate Spc1 activity promotes mitosis while higher levels of Spc1 activity delays mitotic entry. This helped us to elucidate a Spc1-dependent mitotic delay under conditions of Cdc2 hyperactivation previously. It has been reported that *Δwee1* cells with a hyperactive Cdc2 have increased demand for dNTP pools and undergo replication stress (Pai et al., 2019). This piece of information helped us to correlate our increased levels of active Spc1 in *Δwee1* cells to the existing replication stress in them. Another valuable report identifying Wis1 to be a substrate for the kinase Cdc2 (Swaffer et al., 2016) also helped us to hypothesise and later prove that Cdc2-driven regulation of Spc1 function operating in a feedback loop is absolutely Wis1-(MAPKK)-dependent.

**Figure:**
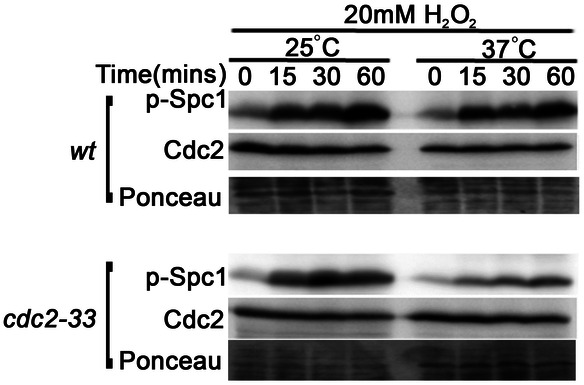
**Cdc2 inactivation: blockage to H_2_O_2_-dependent Spc1 activation.**

**What changes do you think could improve the professional lives of early-career scientists?**

The major hurdle I faced during research at my university was not being able to conduct extensively time consuming experiments because female researchers are not allowed to stay overnight (since it is not a residential campus) hence travelling has a huge impact on the efficiency, planning and meeting targets. Other than that, I have always felt that an independent mind with independent hands should be the best combination for any researcher aiming to set up early careers. But yes, again there is no alternative to hard work. I must mention that I have been fortunate enough to be blessed with all the resources at hand to play around with (not misuse) and I have not been judged for it.

“The major hurdle I faced during research at my university was not being able to conduct extensively time consuming experiments because female researchers are not allowed to stay overnight...”

**What's next for you?**

Working with mice models has remained an urge all through my research career. So if opportunities arise where I will get to learn the basics I would intend to extend my research goals to identifying new therapeutic targets and establishing a link with the already known ones, i.e. p38, Wee1 and 14-3-3.
